# Increasing role of *Anopheles funestus* and *Anopheles arabiensis* in malaria transmission in the Kilombero Valley, Tanzania

**DOI:** 10.1186/1475-2875-13-331

**Published:** 2014-08-24

**Authors:** Dickson W Lwetoijera, Caroline Harris, Samson S Kiware, Stefan Dongus, Gregor J Devine, Philip J McCall, Silas Majambere

**Affiliations:** Environmental Health and Ecological Sciences Thematic Group, Ifakara Health Institute, PO Box 53, Ifakara, Tanzania; Vector Biology Department, Liverpool School of Tropical Medicine, Pembroke Place, Liverpool, L3 5QA UK; Division of Biomedical and Life Sciences, Lancaster University, Bailrigg, Lancaster, LA1 4YW UK; Department of Mathematics, Statistics and Computer Science, Marquette University, Milwaukee, WI 53201-1881 USA; Department of Epidemiology and Public Health, Swiss Tropical and Public Health Institute, Socintr 57, PO Box, CH-4002 Basel, Switzerland; QIMR Berghofer Medical Research Institute, Brisbane, Queensland Australia

**Keywords:** Malaria, *Anopheles*, Transmission, Vector, Surveillance, *Gambiae*, *Arabiensis*, *Funestus*, Season, Insecticide, Susceptibility, EIR, Kilombero, Tanzania

## Abstract

**Background:**

In order to sustain the gains achieved by current malaria control strategies, robust surveillance systems that monitor dynamics of vectors and their roles in malaria transmission over time are essential. This longitudinal study demonstrates the trends in malaria vector dynamics and their relative contribution to malaria transmission in hyperendemic transmission settings in Tanzania.

**Methods:**

The study was conducted in two villages within the Kilombero Valley, in rural Tanzania for five consecutive years (2008–2012). Seventy-two houses were selected per village and each house was sampled for mosquitoes monthly using a CDC light trap. Collected mosquitoes were assessed for species identity and sporozoite infection status using PCR and ELISA, respectively. *Anopheles funestus* and *Anopheles arabiensis* susceptibility to insecticides was assessed using WHO guidelines.

**Results:**

A total of 100,810 malaria vectors were collected, of which 76% were *Anopheles gambiae s. l.* and 24% were *An. funestus*. Of all *An. funestus* samples that amplified with PCR (n = 2,737), 97% were *An. funestus s.s.,* 2% were *Anopheles rivorulum* and 1% *Anopheles leesoni*. Whereas for *An. gambiae s.l.* (n = 8,117), 93% were *An. arabiensis* and 7% were *Anopheles gambiae s.s.* The proportion of *An. gambiae s.s.* identified by PCR (2,924) declined from 0.2% in the year 2008 to undetectable levels in 2012. Malaria transmission intensity significantly decreased from an EIR of 78.14 infectious bites/person/year in 2008 to 35 ib/p/yr in 2011 but rebounded to 226 ib/p/yr in 2012 coinciding with an increased role of *An. funestus* in malaria transmission. Insecticide susceptibility tests indicated high levels of resistance in *An. funestus* against deltamethrin (87%), permethrin (65%), lambda cyhalothrin (74%), bendiocarb (65%), and DDT (66%). Similarly, *An. arabiensis* showed insecticide resistance to deltamethrin (64%), permethrin (77%) and lambda cyhalothrin (42%) in 2014.

**Conclusion:**

The results indicate the continuing role of *An. arabiensis* and the increasing importance of *An. funestus* in malaria transmission, and pyrethroid resistance development in both species. Complementary vector control and surveillance tools are needed that target the ecology, behaviour and insecticide resistance management of these vector species, in order to preserve the efficacy of LLINs.

## Background

Malaria transmission in humans is sustained through vector-human interactions [1] and vector control interventions, such as long-lasting, insecticidal nets (LLINs), aim to break this interaction. Major promotion of LLINs in recent years has resulted in average household ownership rates and usage of LLINs of approximately 42 and 36%, respectively, in sub-Saharan Africa [2]. In mainland Tanzania, a recent report by the Tanzania HIV and Malaria Indicator Survey (THMIS) indicates that above average LLIN ownership and usage (approximately 90 and 66%, respectively) was associated with improved malaria control and overall reduction in malaria prevalence [3].

One outcome of LLIN use is that, by limiting availability of human hosts [4–6], vector species composition in any given area can change considerably after a long period of LLIN use. *Anopheles gambiae sensu stricto, Anopheles arabiensis* and *Anopheles funestus* are the primary malaria vectors in sub-Saharan Africa [7, 8], often occurring sympatrically [9]. *Anopheles gambiae s.s.* is often regarded as the most important vector species across Africa [9–11] and, because of its almost entirely anthropophagic and endophilic behaviour, it is the species that has been targeted most effectively by LLINs.

However, in some locations, populations of *An. gambiae s.s*. have developed insecticide resistance and it continues to be the dominant vector [12, 13]. In other locations, *An. gambiae s.s*. populations have crashed and the relative importance of the remaining vector species has shifted, with *An. arabiensis* becoming the major malaria vector [4, 5, 11].

Since single populations of *An. arabiensis* can exhibit a range of behaviours, biting and resting indoors as well as outdoors and feeding on both humans and animals, interventions that optimally target indoor resting and biting vectors often impact far less on this species [11, 14–17]. The primary vector of the *An. funestus* complex, *An. funestus* is also a very anthropophilic and endophilic mosquito and it too can be a highly efficient malaria vector [10, 18, 19].

Kilombero Valley in southern Tanzania has been subject to a large number of studies on malaria epidemiology, dating back many years, with malaria parasite prevalence rates of up to 70% and an entomological inoculation rate (EIR) of 300 infectious bites per person per year (ib/p/yr) being recorded in the 1990s, the period before the introduction of bed nets [20]. Following the scaling up of untreated nets in the early 2000s [21] and insecticide-treated bed nets (ITNs) and LLINs from 2004 to 2011 [22–24], a continuous decline in malaria vector numbers and malaria transmission has been seen [3, 12]. Although the populations of *An. gambiae s.s.* are significantly dwindling in southern and other parts of Tanzania [12], the remaining populations of *An. arabiensis* and *An. funestus* appears to have shifted their blood-feeding periodicity to optimize their chances to obtain blood meal from their preferred hosts even in the time of low LLIN coverage [25]. It is however suggested that prolonged, widespread use of LLINs is likely to favour outdoor and early biting, either as an expression of the mosquito’s innate phenotypic plasticity or possibly as a heritable, selectable trait that might be expected to increase in frequency [25].

The malaria vector populations in this area are subject to ongoing rigorous monitoring and herein five years of data to the end of 2012 are reported; describing changes in vector species composition and relative abundance, insecticide susceptibility and their contribution to malaria transmission following the years of widespread LLIN use since first introduced in 2004.

## Methods

### Study site

**Figure 1:**
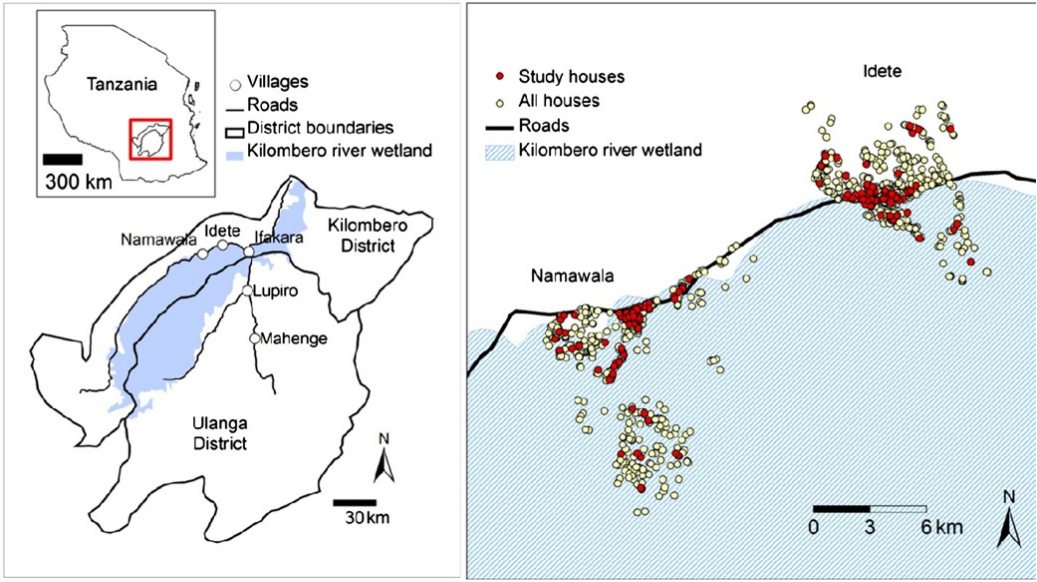
**A map showing sentinel houses for mosquito sampling in Idete and Namwawala villages, within Kilombero Valley, in Kilombero district, Tanzania.**

The study was carried out in Namwawala (8.154425°S and 36.393005°E) and Idete (8.098190°S and 36.510350°E) villages (Figure 1) located in the flood plain of the Kilombero River (8.1°S and 36.6°E) in southeastern Tanzania. The epidemiology of malaria transmission and associated vector species composition within these villages has been documented over many years [21, 25]. Both villages experience an annual rainy season (January-May) and the main crops are rice and maize. Both villages are similar in size (Namwawala = 844 and Idete = 804) and approximately 92% of community members sleep under an ITN or LLIN [12].

### Study design

This study was conducted over five years between January 2008 and December 2012. A total of 72 houses from each village were randomly selected from the Ifakara Health Institute (IHI) Demographic Surveillance System household list [26]. All selected houses were geolocated using a handheld GPS (eTrex, Vista, Garmin, USA). Mosquitoes were sampled in every house each month during 2008, 2011 and 2012 and for six months from January to June in 2009 and July to December in 2010.

### Mosquito sampling and processing

Mosquitoes were sampled using miniature Center for Disease Control (CDC) light traps (model 512, USA). One CDC light trap was used overnight per house, placed 1–1.5 m from the fan above the ground close to the foot end of an occupied bed, and left to run for 12 hours (19.00-07.00) [27, 28]. For every participating house, one LLIN (Olyset, A to Z Textiles Mills, Arusha, Tanzania) was provided to protect the bed occupant where the CDC trap was set. The following morning, CDC light traps were collected and mosquitoes killed using chloroform, and identified in the field using a morphological key [18]. Female mosquitoes were classified as being unfed, partially fed, fully fed or gravid. Subsamples of five mosquitoes from each trap for *An. arabiensis* and *An. funestus* species were individually stored inside a tube containing cotton wool and silica gel beneath for further individual molecular species identification using polymerase chain reaction (PCR) assay for the *An. gambiae* complex [29] and *An. funestus* group [30] and sporozoite infection status using enzyme-linked immunosorbent assay (ELISA) [31] in the laboratory (species identification for the *An. funestus* group did not begin until 2009).

All the sorting information and laboratory analysis results were recorded using designated data collection forms for entomological studies (Kiware *et al.*, unpublished). In addition, variations in malaria transmission by different vector species over time were assessed and compared using the annual EIR calculated by biting rate (total collections/trap nights/year) and the proportion of females infected with sporozoites [32]. Monthly average rainfall data for 2008–2011 were obtained from the Kilombero Valley Teak Company (approximately 15 km from Idete village), and data for year 2012 data were obtained using rain gauges installed in Namwawala village.

### Insecticide susceptibility tests

Following significant increase in *An. funestus* population in 2012, despite extensive usage of LLIN in the study area, it was unclear whether this was due to its reduced susceptibility to the insecticides used in LLINs. The tests were conducted using WHO standard procedures and test kits for adult female mosquitoes of *An. arabiensis* and *An. funestus* [33] in Namwawala villages from January to June 2013. As the confirmatory process, the biossays were repeated in June 2014 for both species.

Five classes of insecticides currently recommended for vector control were tested using discriminating concentrations impregnated in pre-prepared test papers as follows: deltamethrin (0.05%), permethrin (0.75%), lambda cyhalothrin (0.05%), bendiocarb (0.1%), and DDT (4%). Unfed female wild *An. funestus* collected using CDC light traps were used for insecticide exposure bioassays, as recommended by WHO for this difficult-to-colonize species [33]. However, this method is limited by greater variation in susceptibility due to unknown age differences between test mosquitoes, it is simple to carry in the field with minimal infrastructure and test mosquitoes highly representative of the natural population [33].

Prior to exposure, morphologically identified mosquitoes were maintained on 10% glucose solution for at least five hours prior to testing; whereas, for *An. arabiensis*, F1 female mosquitoes two to three days old (recommended age group) were used for bioassays from reared *Anopheles* larvae collected from the breeding habitats in the study sites [34, 35]. Species identification was carried out after bioassays on dead mosquitoes using PCR.

A total of 100 mosquitoes were exposed per discriminating concentration in five replicates of 20 mosquitoes each, and compared to a control with same number of mosquitoes per replicate. In an exposure tube, mosquitoes were held for a total of one hour in intervals of 10, 15, 20, 30, 40, 50, and 60 minutes. After the first hour of exposure, mosquitoes were transferred to non-insecticide treated, clean, holding tubes and observed for a further 20 minutes [33]. After 80 minutes (initial 60 min + further 20 min) of knockdown monitoring, all mosquitoes were transferred to non-insecticide treated, clean, holding tubes and kept for 24 hours and provided with 10% glucose solution, after which mortality was monitored and recorded. All these procedures were performed in the field under average ambient temperatures of 26 ± 2°C and a relative humidity of 78 ± 3% in both bioassay rounds. Percentage knockdown in the observed mosquitoes was recorded immediately for each time interval, and mosquito mortality in each bioassay was expressed as the proportion of dead mosquitoes to total exposed, for each tested insecticide. Execution and interpretation followed recently updated WHO test procedures for insecticide resistance monitoring in malaria vector mosquitoes [33].

### Statistical analyses

Only data pertaining to *An. gambiae s.l.* and *An. funestus* were analysed, using SPSS version 20 (SPSS Inc, Chicago, USA). Data were fitted with generalized linear models (GLMs) using a negative binomial distribution with log-link function, and relative rates (RR) with 95% confidence intervals calculated to estimate yearly mean mosquito catches, relative to the reference year. Species (*An. gambiae s.l.* and *An. funestus)* were treated as predictors and total number of mosquitoes as a dependent variable; the statistical differences in dependent variables was evaluated as a function of villages (Idete and Namwawala), seasons (wet and dry) and years (2008–2012).

Insecticide susceptibility test biossay data were considered for each diagnostic concentration and year of testing. Mortality was calculated as the percentage of mosquitoes dead post 24 hours’ exposure to insecticide, and the results were assessed according to WHO testing procedure for insecticide resistance monitoring in malaria vectors [33]. Mortality rates between 98 and 100% indicate full susceptibility, 90-97% is suggestive of resistance and requires further investigation, and mortality rates less than 90% confirm the existence of resistance.

### Ethical considerations

The study approval was granted by the Ifakara Health Institutional Review Board (IHRDC/IRB/No.A-32) and the National Institute of Medical Research (NIMR/HQ/R.8a/Vol. IX/764). On first visiting each house, the benefits and possible risks associated with the study were explained to the house occupants and informed consent to proceed was requested. After consenting, the head of the house was asked to sign two copies of the informed consent forms, (retained by the head of the house and the study investigator).

## Results

### Relative abundance of malaria vector species

During the five consecutive years of sampling with CDC light traps in sentinel houses, a total of 100,810 malaria vectors were collected of which 76% were *Anopheles gambiae sensu lato* and 24% were *An. funestus*. In each of the first four years (2008–2011), the proportion of *An. gambiae s.l.* was significantly higher than *An. funestus* in both study villages (*p* <0.0001): proportions in total catches in Namwawala were 94% (40,028) and 6% (2,398), and in Idete were 87% (24,869) and 13% (3,730) for *An. gambiae s.l.* and *An. funestus*, respectively. However, in 2012, the proportion of total catch of *An. funestus* was significantly higher than *An. gambiae s.l.* in both villages: 42% (6,622) and 58% (8,953) in Namwawala and 35% (4,479) and 65% (8,447) in Idete for *An. gambiae s.l.* and *An. funestus*, respectively, (RR (95% CI) = 1.35 (1.23-1.49), *p* <0.0001).

A total of 8,117 *An. gambiae s.l.* were successfully identified by PCR and comprised 93% *An. arabiensis* (n = 7,549) and 7% *An. gambiae s.s.* (n = 568). The relative proportions of the species were similar in Idete (*An. arabiensis* 96% (n = 3,610), *An. gambiae s.s.* 4% (n = 151) and in Namwawala 90% (n = 3,900) *An. arabiensis*, 10% (n = 456) *An. gambiae s.s*.. However, the relative proportion between the two sibling species was changing over time, with significant decrease of *An. gambiae s.s.* from 14% (409/2,924 ) in year 2008 to disappearance 0% (0/1,362 ) in year 2012, compared to *An. arabiensis* increasing from 86% in 2008 to 100% in 2012 (Table 1).

**Table 1:** **Malaria vector composition, sporozoite prevalence (S), biting rate (B) and entomological inoculation (EIR) for** ***Anopheles gambiae s.s., Anopheles arabiensis*** **and** ***Anopheles funestus*** **and their overall estimated yearly contribution to malaria transmission from year 2008–2012 in the study area**

Species	2008	2009/10	2011	2012
***An. gambiae complex*** **sibling species proportion**				
*An. gambiae s.s.*	0.14	0.15	0.002	0
*An. arabiensis*	0.86	0.85	0.998	1
No. of PCR amplifications	2,924	1,307	2,542	1,362
***An. funestus*** **group sibling species proportion**				
*An. funestus s.s.*	-	0.887	0.956	1
*An. rivulorum*	-	0.013	0.021	0
*An. leesoni*	-	0	0.023	0
*An. parensis*	-	0	0.001	0
No. of PCR amplifications	-	330	880	1,527
**Sporozoite prevalence (S;%)**				
*An. gambiae s.s.*	1.18	0.04	0	0
*An. arabiensis*	0.16	0.36	0.07	1.47
*An. funestus*	1.71	0	0.43	2.20
**Biting rate (B; b/p/n)**				
*An. gambiae s.s.*	8.52	6.05	0.04	0
*An. arabiensis*	52.37	35.51	59.74	20.70
*An. funestus*	1.74	12.84	10.09	14.31
**Entomological Inoculation Rate (EIR; ib/p/y)**				
*An. gambiae s.s.*	36.70	1.61	0	0
*An. arabiensis*	30.58	55.51	15.17	110.90
*An. funestus*	10.86	0	15.58	115.10
**Total**	**78.14**	**57.12**	**31.05**	**226.0**

Note: Sporozoite prevalence = Number of positive sporozoite mosquitoes/total tested; Biting rate = Total collections/trap nights/calibration factor, 0.3 for *An. gambiae* complex, and 0.68 for *An. funestus* [21]; EIR = S × B × 365.

Of the 2,737 *An. funestus* samples that were identified by PCR, 97% were *An. funestus s.s.* (n = 2,655), 2% were *Anopheles rivorulum* (n = 55) and 1% *Anopheles leesoni* (n = 27). The species composition of *An. funestus* in Idete was 98% (n = 1,554) *An. funestus s.s,* 1.5% (n = 23) *An. rivorulum* and 0.4% (n = 6) *An. leesoni.* In Namwawala it was 98% (n = 1,133) *An. funestus s.s.,* 0.6% (n = 7) *An. rivorulum* and 1.2% (n = 14) *An. leesoni,* (Table 1).

### Seasonal variation in vector abundance

**Figure 2:**
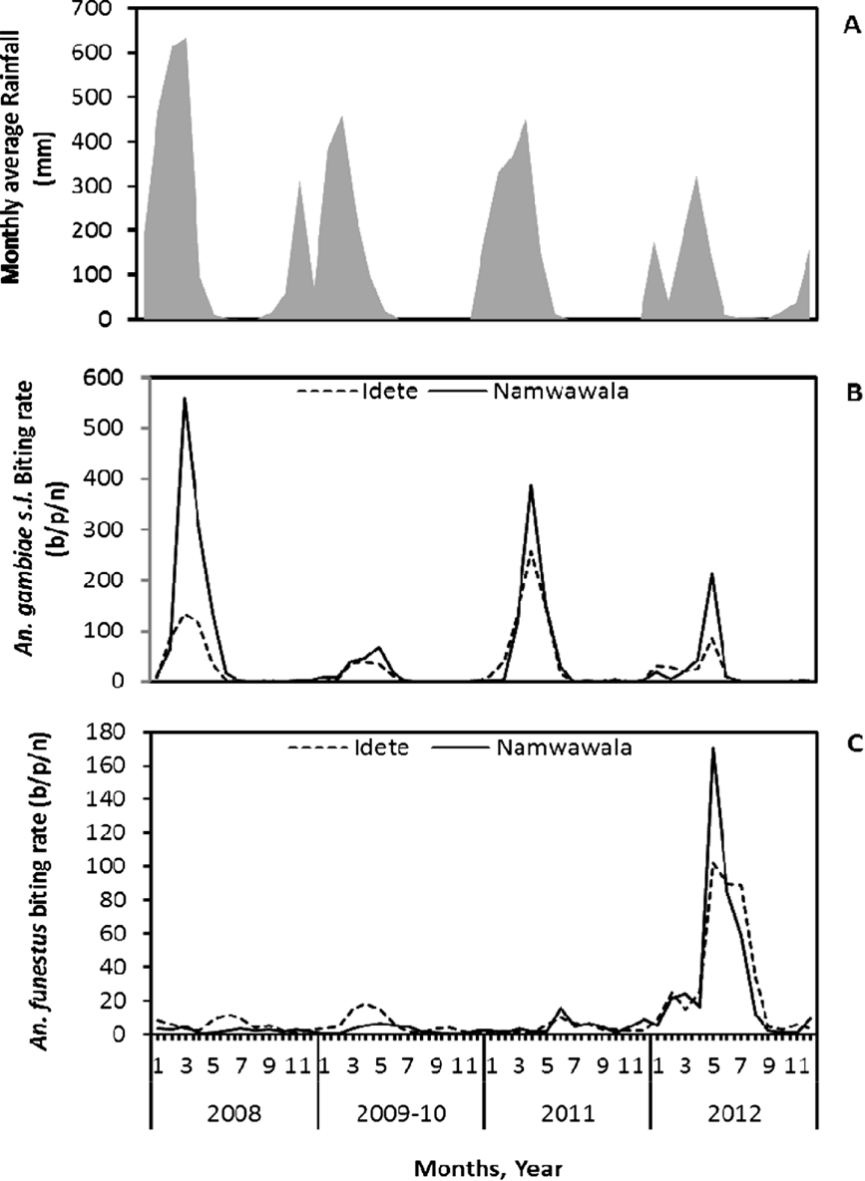
**Monthly average rainfall in the Kilombero Valley. (A)** estimated using CDC monthly biting rates, adjusted by dividing by species-specific relative efficiency of 0.3 and 0.68 for *An. gambiae s.l.* **(B)** and *An. funestus* **(C)**, respectively [21], in Idete and Namwawala villages over time.

During the study, the period from January to May was categorized as the wet season, receiving an average (+SD) of rainfall of 281 + 178 mm/month, and June-December as the dry season, with an average of rainfall of 24 + 66 mm/month (Figure 2). The abundance of both *An. gambiae s.l.* and *An. funestus* peaked in the wet season in both villages. The mean number (+SD) of *An. gambiae s.l.* caught per trap per night during the wet season was 19 + 48 and 32 + 110, whereas in the dry season it decreased to 0.86 + 5.7 and 1.1 + 5.8 at Idete and Namwawala, respectively. Furthermore, *An. gambaie s.s*. was only present in the wet season in the first three years (2008-2009/10) before its disappearance in 2011/12, compared to its sibling species *An. arabiensis*, which was found to exist in both season, similar to *An. funestus s.s.*, a dominating member of *An. funestus* group.

The mean number of *An. funestus* per trap per night in the wet and dry season of the first four years of study (2008–2011) was consistently similar in both villages. In the wet season, the mean catches (+SD) were 1.23 + 4.7 in 2008, 2.15 + 7.5 in 2009/10, 0.64 + 1.9 in 2011 compared to 1.15 + 5.2, 0.77 + 4.3 and 1.62 + 5.52 of the respective years in the dry season. In 2012, the mean catch of *An. funestus*, both in wet and dry seasons, was approximately six times significantly higher than in the previous years (*p* <0.0001): 11.75 + 45.8 and 8.3 + 25.6 of wet and dry season, respectively.

### Malaria transmission

A total of 10,138 individual mosquitoes (530 *An. gambiae s.s.,* 7,130 *An. arabiensis* and 2,478 *An. funestus s.s.*) were screened for *Plasmodium falciparum* sporozoites of which 75 were positive (0.74% sporozoite prevalence). Although *An. gambiae s.s*. was the major malaria vector with a sporozoite prevalence of 1.18% in 2008, its dominance decreased with time to zero in 2011 and 2012, following its control to undetectable levels. Conversely, the importance of *An. arabiensis* and *An. funestus s.s* was increasing with time from a sporozoite prevalence of 0.16% in 2008 to 1.47% in 2012 for *An. arabiensis*, and from 1.71% in 2008 to 2.2% in 2012 for *An. funestus s.s.*

Similarly, the EIR of *An. gambiae s.s.* decreased drastically from 30.70 ib/p/yr in 2008 to 0 ib/p/yr in 2012, whereas those of *An. arabiensis* increased approximately four times from 30.58 in 2008 to 110.9 in 2012 and that of *An. funestus s.s.* increased 11 times from 10.86 in 2008 to 115.10 in 2012.

Overall, the level of malaria transmission in the study villages markedly decreased with time from an EIR of 78.14 ib/p/yr in 2008 to 31.05 ib/p/yr in 2011 but overwhelmingly increased to 226 ib/p/yr in 2012, approximately seven times more than in the previous year (Table 1).

### *Anopheles arabiensis and Anopheles funestus*insecticide susceptibility tests

**Figure 3:**
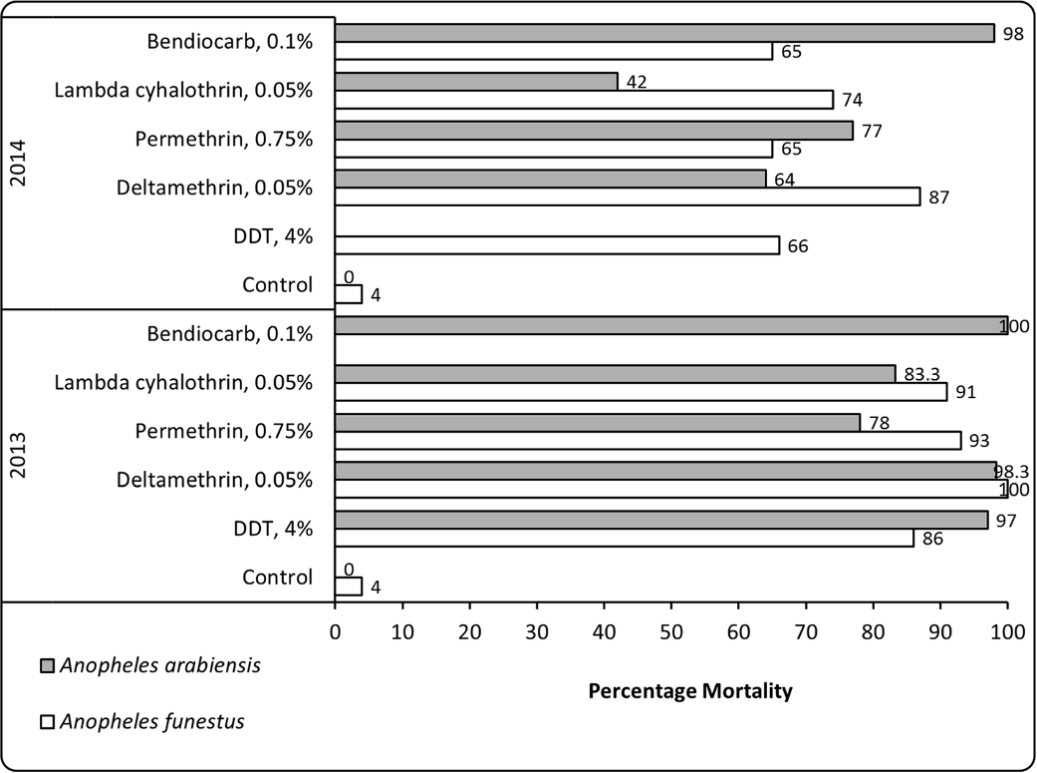
**Results of WHO bioassay test for insecticide susceptibility status of wild female** ***Anopheles funestus*** **(white bars) and** ***Anopheles arabiensis*** **(grey bars) from the study sites in the Kilombero Valley, Tanzania, in January 2013 and June 2014.** The graph shows percentage 24 hours mortality rate after a one-hour exposure to the WHO diagnostic doses of insecticide. The minimum sample size for these assays was 100.

In the WHO bioassay testing, as the results indicated (Figure 3), *An. funestus* was fully susceptible to deltamethrin (100% mortality) with reduced susceptibility to permethrin (93%), and lambda cyhalothrin (91%) and confirmed resistance to DDT (86%) in year 2013. In 2014, *An. funestus* was resistant to permethrin (65%), lambda cyhalothrin (74%), bendiocarb (65%), and even to deltamethrin (87%) to which it was fully susceptible in 2013. Mortality in control tubes was 4% in both testing rounds. All tested mosquitoes were amplified as *An. funestus,* using PCR.

In year 2013, *An. arabiensis* was fully susceptible to bendiocarb (100% mortality) and deltamethrin (98.3%), reduced susceptibility against DDT (97%), and confirmed resistance to permethrin (83.3%) and lambda cyhalothrin (78%), with a control mortality of 0% across all test concentrations. Similar levels of resistance were maintained across tested diagnostic concentrations in year 2014, whereby the mosquitoes were fully susceptible to bendiocarb (98% mortality) and resistant to deltamethrin (64%), permethrin (77%), and lambda cyhalothrin (42%), with a control mortality of 0% across all test concentrations.

## Discussion

This study provides substantial information on malaria vector dynamics and their contribution to malaria transmission in rural southern Tanzania over a five year period. Consistent with other studies, which have documented a shift in malaria vector composition and a change in malaria transmission dynamics seemingly as a result of extensive use of LLINs [4, 5, 36], this study reports a steady decrease to undetectable levels of *An. gambiae s.s.* with steady increase in the proportion of its sibling species *An. arabiensis* and a surge in the abundance of *An. funestus s.s.* in year 2012.

*Anopheles gambiae s.s.* prefers to feed and rest inside houses. This makes it more vulnerable to insecticides applied to nets (LLINs) and walls (indoor residual spraying (IRS)) while *An. arabiensis*, with its opportunistic feeding behaviour both on humans and animals [12, 32] and its potential to rest outside human dwellings, make it less affected by LLINs. Although, lack of outdoor mosquito collections was a major limitation of this study in explaining the shift in biting periodicity and outdoor biting, it has been recently documented elsewhere that *An. arabiensis* and *An. funestus* [25, 37] display a behavioural avoidance to contact LLINs by feeding outdoors in early part of the evening which might increase its chance to survive current interventions.

A significant increase in *An. funestus* abundance and EIR in 2012 is demonstrated. This shift poses great concern in malaria control efforts due to its efficiency in transmitting malaria. Historically the control of *An. funestus s.s.* was successful through extensive IRS, taking advantage of its highly anthropophagic and endophilic behaviour, using dieldrin in Pare, Taveta, northern Tanzania [38, 39] Malindi on the coast of Kenya, using DDT [40] as well as in South Africa [41]. This is partly because they spend a longer time on insecticide-treated materials [42]. However, the vector eventually resurged six years later due to a lack of IRS programme continuity and consolidation [40, 43]. A similar scenario was expected in this particular region, where usage of LLINs is high [3, 44].

The steady increase in *An. funestus* population density, despite extensive usage of LLINs in the study area, may be due to its reduced susceptibility to the insecticides used in LLINs. Recent findings from western Kenya have demonstrated similar phenomenon of resurging *An. funestus* populations, chiefly being due to resistance development to the pyrethroids used in LLINs [45].

Preliminary findings from this study demonstrated high resistance of *An. funestus* and *An. arabiensis* to pyrethroids, deltamethrin, lambda cyhalothrin and permethrin, used in Olyset LLINs, distributed in the study area in June 2011 [3]. Overall, there was great variation of the resistance status between 2013 and 2014 in both species tested; however, the variation was surprisingly huge in *An. funestus* than *An. arabiensis*, which might be due to inconsistency in unknown age of the used wild mosquito females [33].

Due to the absence of organochlorine insecticide DDT and carbamate insecticide bendiocarb deployment for malaria vector control in the study area, the source of resistance in mosquitoes to these insecticides remains unknown. Although not tested in this particular study, pyrethroid (DDT and pyrethroid) carbamate cross-resistance was considered to be a probable cause of *An. funestus* resistance to DDT and bendiocarb, respectively, which has been proved to exist in malaria vectors elsewhere [46, 47]. In addition, the continuous and illegitimate use of DDT as a pesticide in agriculture in the region might have contaminated malaria vector breeding habitats and caused physiological resistance in mosquitoes [48].

Pyrethroid resistance in both species has been documented in multiple countries and regions of East Africa [45, 49, 50], southern Africa [51–54] and West Africa [55–57]. Further detailed studies are urgently required to establish current vector control operational impacts associated with this level of resistance. These findings suggest an increased contribution of these vectors to malaria transmission and hence great threat to the future use of LLINs in controlling these vectors.

The other probable cause for the observed increase in *An. funestus* population in this study area, which requires further investigation, might be a shift of *An. funestus* to outdoor and early evening and daytime biting behaviours, which increase their chances to survive and reproduce by feeding on unprotected humans, as recently documented *An. funestus* behaviours in Benin [58] and Senegal [59], West Africa.

In this study, both *An. funestus* and *An. gambiae s.l.* vector abundance varied with season. Increases in *An. gambiae s.l.* densities are facilitated by a wide range of ephemeral, sunlit, breeding habitats, such as hoof prints, rice puddles and ground depressions created during the rainy season [18, 60]. The temporary nature of these habitats tends to reduce predation rate but also allows quick development of the juvenile stages, which results in *An. gambiae s.l.* dominating during the rainy season [18]. On the contrary, *An. funestus* prefer vegetated semi-permanent and permanent breeding habitats, such as swamps and large ponds [18]. *Anopheles funestus* remained at a reasonable and detectable density across the rainy and dry seasons in the study areas and were significantly more abundant than *An. gambiae s.l.* in the dry season, probably due to their breeding habitat stability against desiccation [61].

Irrespective of seasonal variation in vector abundance, *An. funestus s.s.*, *An. gambiae s.s.* and *An. arabiensis* were all-important malaria vectors in the study area [12]. Despite high abundance of *An. arabiensis* and a higher EIR between 2008 and 2010, *An. funestus* contributed a relatively higher or equal EIR in 2011 and 2012. Historically, *An. funestus* has displayed high sporozoite prevalence [62] similar to that observed in this study and in a recent study conducted in neighbouring villages within the valley (Kaindoa *et al.,* unpublished). This trend of increase in abundance and high sporozoite prevalence of *An. funestus* has been also observed in Asembo district, western Kenya [45] and so appears to represent a trend across several regions of East Africa.

The huge increase in potential malaria transmission in 2012 (EIR = 226) coincided with an increase in abundance and sporozoite rates in *An. funestus* as it did in a neighbouring village in the valley (EIR = 467) (Kaindoa *et al.*, unpublished). The substantial increase in *An. funestus* and its reduced susceptibility to pyrethroids poses a serious threat that needs attention from vector control stakeholders. A separate study in West Africa also reported a rebound in malaria transmission partly being caused by resistance development in *An. gambiae* to pyrethroids [63].

A previous study has shown that despite high coverage and usage of LLINs, a high proportion of mosquitoes still enter houses [64]. Therefore, the increase in *An. funestus*, particularly in the dry season, is likely to exacerbate the problem. Therefore, new strategies to address resistance and outdoor biting behaviour in the early part of the evening as displayed by *An. funestus* and *An. arabiensis* are required. This can be achieved through improving the LLINs; for instance, recent development of nets which can target multiple resistant mosquitoes, Olyset® Plus [65], and by targeting vectors while outdoors using non-resistant compounds, either through larval source management in the dry season via autodissemination of insect juvenile hormone, e.g., pyriproxyfen [66, 67], or by mosquito sterilization with pyriproxyfen [68], and killing them with toxic sugar-baited traps [69], non-chemical electric grid [70] and odour-baited traps [71].

## Conclusion

This study shows that *An. funestus* and *An. arabiensis* are important malaria vectors sustaining malaria transmission, with a substantial increase in *An. funestus* and drastic reduction in *An. gambiae s.s.* in the year 2012. Malaria transmission significantly declined from 2008 to 2011 and rebounded in 2012 coinciding with an increased role of *An. arabiensis* and *An. funestus* in malaria transmission. Although fully susceptible to deltamethrin, *An. arabiensis* and *An. funestus* were found to be resistant and with reduced susceptibility to permethrin pyrethroid used for LLINs, respectively. These findings call for complementary vector control tools, robust vector surveillance systems and an insecticide resistance management plan to complement and preserve the efficacy of LLINs.

## References

[1] Bruce-Chwatt LJ, Garret-Jones C, Weitz B (1966). Ten year study (1955–64) of host selection by Anopheline mosquitoes. Bull World Health Organ.

[2] WHO (2013). World Malaria Report.

[3] Tanzania Commission for AIDS ZAC, National Bureau of Statistics, Office of the Chief Government Statistician, ICF International Calverton MU (2013). Tanzania HIV/AIDS and Malaria Indicator Survey 2011–12.

[4] Bayoh MN, Mathias DK, Odiere MR, Mutuku FM, Kamau L, Gimnig JE, Vulule JM, Hawley WA, Hamel MJ, Walker ED (2010). *Anopheles gambiae:* historical population decline associated with regional distribution of insecticide-treated bed nets in western Nyanza Province. Kenya Malar J.

[5] Mwangangi JM, Mbogo CM, Orindi BO, Muturi EJ, Midega JT, Nzovu J, Gatakaa H, Githure J, Borgemeister C, Keating J (2013). Shifts in malaria vector species composition and transmission dynamics along the Kenyan coast over the past 20 years. Malar J.

[6] Okumu FO, Kiware SS, Moore SJ, Killeen GF (2013). Mathematical evaluation of community level impact of combining bed nets and indoor residual spraying upon malaria transmission in areas where the main vectors are *Anopheles arabiensis* mosquitoes. Parasit Vectors.

[7] Mzilahowa T, Hastings IM, Molyneux ME, McCall PJ (2012). Entomological indices of malaria transmission in Chikhwawa district. Southern Malawi Malar J.

[8] Sinka ME, Bangs MJ, Manguin S, Rubio-Palis Y, Chareonviriyaphap T, Coetzee M, Mbogo CM, Hemingway J, Patil AP, Temperley WH (2012). A global map of dominant malaria vectors. Parasit Vectors.

[9] Coetzee M, Craig M, Le Sueur D (2000). Distribution of African malaria mosquitoes belonging to the *Anopheles gambiae* complex. Parasitol Today.

[10] Gillies M, Coetzee M (1987). A Supplement of the Anophelinae of Africa South of the Sahara (Afrotropical region).

[11] Russell TL, Lwetoijera DW, Maliti D, Chipwaza B, Kihonda J, Charlwood D, Smith TA, Lengeler C, Mwanyangala MA, Nathan R, Knols BGJ, Takken W, Killeen GF (2010). Impact of promoting longer-lasting insecticide treatment of bed nets upon malaria transmission in a rural Tanzanian setting with pre-existing high coverage of untreated nets. Malar J.

[12] Corbel V, N'Guessan R, Brengues C, Chandre F, Djogbenou L, Martin T, Akogbeto M, Hougard JM, Rowland M (2007). Multiple insecticide resistance mechanisms in *Anopheles gambiae* and *Culex quinquefasciatus* from Benin, West Africa. Acta Trop.

[13] N'Guessan R, Corbel V, Akogbeto M, Rowland M (2007). Reduced efficacy of insecticide-treated nets and indoor residual spraying for malaria control in pyrethroid resistance area, Benin. Emerg Infect Dis.

[14] Muriu SM, Muturi EJ, Shililu JI, Mbogo CM, Mwangangi JM, Jacob BG, Irungu LW, Mukabana RW, Githure JI, Novak RJ (2008). Host choice and multiple blood feeding behaviour of malaria vectors and other anophelines in Mwea rice scheme. Kenya Malar J.

[15] Tirados I, Costantini C, Gibson G, Torr SJ (2006). Blood-feeding behaviour of the malarial mosquito *Anopheles arabiensis*: implications for vector control. Med Vet Entomol.

[16] Kitau J, Oxborough RM, Tungu PK, Matowo J, Malima RC, Magesa SM, Bruce J, Mosha FW, Rowland MW (2012). Species shifts in the *Anopheles gambiae* complex: do LLINs successfully control *Anopheles arabiensis*?. PLoS One.

[17] White G, Magayuka SA, Boreham PFL (1972). Comparative studies on sibling species of the *Anopheles gambiae* Giles complex (Dipt., Culicidae): bionomics and vectorial activity of species A and species B at Segera, Tanzania. Bull Entomol Res.

[18] Gillies M, de Meillon B (1968). The Anophelini of Africa South of the Sahara (Ethiopian Zoogeographical Region).

[19] Mendis C, Jacobsen JL, Gamage-Mendis A, Bule E, Dgedge M, Thompson R, Cuamba N, Barreto J, Begtrup K, Sinden RE, Hogh B (2000). *Anopheles arabiensis* and *An. funestus* are equally important vectors of malaria in Matola coastal suburb of Maputo, southern Mozambique. Med Vet Entomol.

[20] Smith T, Charlwood JD, Kihonda J, Mwankusye S, Billingsley P, Meuwissen J, Lyimo E, Takken W, Teuscher T, Tanner M (1993). Absence of seasonal variation in malaria parasitaemia in an area of intense seasonal transmission. Acta Trop.

[21] Killeen G, Tami A, Kihonda J, Okumu F, Kotas M (2007). Cost-sharing strategies combining targeted public subsidies with private-sector delivery achieve high bednet coverage and reduced malaria transmission in Kilombero Valley, southern Tanzania. BMC Infect Dis.

[22] Alba S, Hetzel MW, Nathan R, Alexander M, Lengeler C (2011). Assessing the impact of malaria interventions on morbidity through a community-based surveillance system. Int J Epidemiol.

[23] Mulligan JA, Yukich J, Hanson K (2008). Costs and effects of the Tanzanian national voucher scheme for insecticide-treated nets. Malar J.

[24] Renggli S, Mandike R, Kramer K, Patrick F, Brown NJ, McElroy PD, Rimisho W, Msengwa A, Mnzava A, Nathan R (2013). Design, implementation and evaluation of a national campaign to deliver 18 million free long-lasting insecticidal nets to uncovered sleeping spaces in Tanzania. Malar J.

[25] Russell T, Govella N, Azizi S, Drakeley C, Kachur SP, Killeen G (2011). Increased proportions of outdoor feeding among residual malaria vector populations following increased use of insecticide-treated nets in rural Tanzania. Malar J.

[26] Schellenberg JRMA, Abdulla S, Nathan R, Mukasa O, Marchant TJ, Kikumbih N, Mushi AK, Mponda H, Minja H, Mshinda H (2001). Effect of large-scale social marketing of insecticide-treated nets on child survival in rural Tanzania. Lancet.

[27] Lines JD, Curtis CF, Wilkes TJ, Njunwa KJ (1991). Monitoring human-biting mosquitoes (Diptera: Culicidae) in Tanzania with light-traps hung beside mosquito nets. Bull Entomol Res.

[28] Mboera LE, Kihonda J, Braks MA, Knols BG (1998). Short report: influence of centers for disease control light trap position, relative to a human-baited bed net, on catches of *Anopheles gambiae* and *Culex quinquefasciatus* in Tanzania. Am J Trop Med Hyg.

[29] Scott JA, Brogdon WG, Collins FH (1993). Identification of single specimens of the *Anopheles gambiae* complex by the polymerase chain-reaction. Am J Trop Med Hyg.

[30] Koekemoer LL, Kamau L, Hunt RH, Coetzee M (2002). A cocktail polymerase chain reaction assay to identify members of the *Anopheles funestus* (diptera: culicidae) group. Am J Trop Med Hyg.

[31] Burkot T, Williams J, Schneider I (1984). Identification of *Plasmodium falciparum* infected mosquitoes by a double antibody enzyme-linked immunosorbent assay. Am J Trop Med Hyg.

[32] Kelly-Hope LA, McKenzie FE (2009). The multiplicity of malaria transmission: a review of entomological inoculation rate measurements and methods across sub-Saharan Africa. Malar J.

[33] WHO (2013). Test Procedures for Insecticide Resistance Monitoring in Malaria Vector Mosquitoes.

[34] Chouaibou MS, Chabi J, Bingham GV, Knox TB, N’Dri L, Kesse NB, Bonfoh B, Jamet HV (2012). Increase in susceptibility to insecticides with aging of wild *Anopheles gambiae* mosquitoes from Côte d’Ivoire. BMC Infect Dis.

[35] Jones CM, Sanou A, Guelbeogo WM, Sagnon N, Johnson P, Ranson H (2012). Aging partially restores the efficacy of malaria vector control in insecticide-resistant populations of Anopheles gambiae s.l. from Burkina Faso. Malar J.

[36] Derua YA, Alifrangis M, Hosea KM, Meyrowitsch DW, Magesa SM, Pedersen EM, Simonsen PE (2012). Change in composition of the *Anopheles gambiae* complex and its possible implications for the transmission of malaria and lymphatic filariasis in north-eastern Tanzania. Malar J.

[37] Wilkes TJ, Matola YG, Charlwood JD (1996). *Anopheles rivulorum*, a vector of human malaria in Africa. Med Vet Entomol.

[38] Gillies MT, Smith A (1960). Effect of a residual house-spraying campaign on species balance in the *Anopheles funestus* group: the replacement of *Anopheles gambiae* Giles with *Anopheles rivulorum leesoni*. Bull Entomol Res.

[39] Smith A (1966). Malaria in the Taveta area of Kenya and Tanzania. Part IV. Entomological findings six years after the spraying period. East Afr Med J.

[40] Gillies MT, Furlong M (1964). An investigation into the behaviour of *Anopheles parensis* Gillies at Malindi on the coast of Kenya. Bull Entomol Res.

[41] Sharp BL, Kleinschmidt I, Streat E, Maharaj R, Barnes KI, Durrheim DN, Ridl FC, Morris N, Seocharan I, Kunene S, La Grange JJP, Mthembu JD, Maartens F, Martin CL, Barreto A (2007). Seven years of regional malaria control collaboration - Mozambique, South Africa, and Swaziland. Am J Trop Med Hyg.

[42] Davidson G (1953). Experiments on the effects of residual insecticides in houses against *Anopheles gambiae* and *An. funestus*. Bull Entomol Res.

[43] The malERA Consultative Group on Vector Control (2011). A research agenda for malaria eradication: vector control. PLoS Med.

[44] Koenker HM, Yukich JO, Mkindi A, Mandike R, Brown N, Kilian A, Lengeler C (2013). Analysing and recommending options for maintaining universal coverage with long-lasting insecticidal nets: the case of Tanzania in 2011. Malar J.

[45] McCann RS, Ochomo O, Bayoh N, Vulule JM, Gimnig JE, Walker ED (2014). Reemergence of *Anopheles funestus* as a vector of *Plasmodium falciparum* in western Kenya after long-term implementation of insecticide-treated bed nets. Am J Trop Med Hyg.

[46] Brooke B, Kloke G, Hunt R, Koekemoer L, Tem E, Taylor M, Small G, Hemingway J, Coetzee M (2001). Bioassay and biochemical analyses of insecticide resistance in southern African *Anopheles funestus* (Diptera: Culicidae). Bull Entomol Res.

[47] Protopopoff N, Matowo J, Malima R, Kavishe R, Kaaya R, Wright A, West PA, Kleinschmidt I, Kisinza W, Mosha FW (2013). High level of resistance in the mosquito *Anopheles gambiae* to pyrethroid insecticides and reduced susceptibility to bendiocarb in north-western Tanzania. Malar J.

[48] Nkya TE, Akhouayri I, Kisinza W, David J-P (2013). Impact of environment on mosquito response to pyrethroid insecticides: facts, evidences and prospects. Insect Biochem Molec Biol.

[49] Matambo T, Abdalla H, Brooke B, Koekemoer L, Mnzava A, Hunt R, Coetzee M (2007). Insecticide resistance in the malarial mosquito *Anopheles arabiensis* and association with the kdr mutation. Med Vet Entomol.

[50] Morgan JC, Irving H, Okedi LM, Steven A, Wondji CS (2010). Pyrethroid resistance in an *Anopheles funestus* population from Uganda. PLoS One.

[51] Hargreaves K, Hunt RH, Brooke BD, Mthembu J, Weeto MM, Awolola TS, Coetzee M (2003). *Anopheles arabiensis* and *An. quadriannulatus* resistance to DDT in South Africa. Med Vet Entomol.

[52] Casimiro S, Coleman M, Mohloai P, Hemingway J, Sharp B (2006). Insecticide resistance in *Anopheles funestus* (Diptera: Culicidae) from Mozambique. J Med Entomol.

[53] Kloke RG, Nhamahanga E, Hunt RH, Coetzee M (2011). Vectorial status and insecticide resistance of *Anopheles funestus* from a sugar estate in southern Mozambique. Parasit Vectors.

[54] Wondji CS, Coleman M, Kleinschmidt I, Mzilahowa T, Irving H, Ndula M, Rehman A, Morgan J, Barnes KG, Hemingway J (2012). Impact of pyrethroid resistance on operational malaria control in Malawi. Proc Natl Acad Sci.

[55] Okoye PN, Brooke BD, Koekemoer LL, Hunt RH, Coetzee M (2008). Characterisation of DDT, pyrethroid and carbamate resistance in *Anopheles funestus* from Obuasi, Ghana. Trans R Soc Trop Med Hyg.

[56] Ranson H, Guessan R, Lines J, Moiroux N, Nkuni Z, Corbel V (2011). Pyrethroid resistance in African anopheline mosquitoes: what are the implications for malaria control?. Trends Parasitol.

[57] Djouaka R, Irving H, Tukur Z, Wondji CS (2011). Exploring mechanisms of multiple insecticide resistance in a population of the malaria vector *Anopheles funestus* in Benin. PLoS One.

[58] Moiroux N, Gomez MB, Pennetier C, Elanga E, Djènontin A, Chandre F, Djègbé I, Guis H, Corbel V (2012). Changes in *Anopheles funestus* biting behavior following universal coverage of long-lasting insecticidal nets in Benin. J Infect Dis.

[59] Sougoufara S, Diédhiou SM, Doucouré S, Diagne N, Sembène PM, Harry M, Trape J-F, Sokhna C, Ndiath MO (2014). Biting by *Anopheles funestus* in broad daylight after use of long-lasting insecticidal nets: a new challenge to malaria elimination. Malar J.

[60] Minakawa N, Sonye G, Mogi M, Yan G (2004). Habitat characteristics of *Anopheles gambiae s.s.* larvae in a Kenyan highland. Med Vet Entomol.

[61] Charlwood JD, Vij R, Billingsley PF (2000). Dry season refugia of malaria-transmitting mosquitoes in a dry savannah zone of east Africa. Am J Trop Med Hyg.

[62] Charlwood J, Smith T, Kihonda J, Heiz B, Billingsley P, Takken W (1995). Density independent feeding success of malaria vectors (Diptera: Culicidae) in Tanzania. Bull Entomol Res.

[63] Trape J, Tall A, Diagne N, Ndiath O, Ly AB, Faye J, Dieye-Ba F, Roucher C, Bouganali C, Badiane A, Sarr FD, Mazenot C, Touré-Baldé A, Raoult D, Druilhe P, Puijalon OM, Rogier C, Sokhna C (2011). Malaria morbidity and pyrethroid resistance after the introduction of insecticide -treated bednets and artemisinin-based combination therapies: a longitudinal study. Lancet Infect Dis.

[64] Gatton ML, Chitnis N, Churcher T, Donnelly MJ, Ghani AC, Godfray HCJ, Gould F, Hastings I, Marshall J, Ranson H, Rowland M, Shaman J, Linday SW (2013). The importance of mosquito behavioural adaptations to malaria control in Africa. Evolution.

[65] Pennetier C, Bouraima A, Chandre F, Piameu M, Etang J, Rossignol M, Sidick I, Zogo B, Lacroix M-N, Yadav R (2013). Efficacy of Olyset® Plus, a new long-lasting insecticidal net incorporating permethrin and piperonil-butoxide against multi-resistant malaria vectors. PLoS One.

[66] Devine GJ, Zamora Perea E, Killeen GF, Stancil JD, Clark SJ, Morrison AC (2009). Using adult mosquitoes to transfer insecticides to *Aedes aegypti* larval habitats. Proc Natl Acad Sci U S A.

[67] Lwetoijera DW, Harris C, Kiware S, Dongus S, Devine GJ, McCall PJ, Majambere S (2014). Effective autodissemination of pyriproxyfen to breeding sites by the exophilic malaria vector *Anopheles arabiensis* in semi-field settings in Tanzania. Malar J.

[68] Lwetoijera DW, Harris C, Kiware SS, Killeen GF, Dongus S, Devine GJ, Majambere S (2014). Comprehensive sterilization of malaria vectors using pyriproxyfen: a step closer to malaria elimination. Am J Trop Med Hyg.

[69] Muller GC, Schlein Y (2008). Efficacy of toxic sugar baits against adult cistern-dwelling *Anopheles claviger*. Trans R SocTrop Med Hyg.

[70] Majambere S, Masue D, Mlacha Y, Govella NJ, Magesa SM, Killeen GF (2013). Advantages and limitations of commercially available electrocuting grids for studying mosquito behaviour. Parasit Vectors.

[71] Matowo NS, Moore J, Mapua S, Madumla EP, Moshi IR, Kaindoa EW, Mwangungulu SP, Kavishe DR, Sumaye RD, Lwetoijera DW, Okumu FO (2013). Using a new odour-baited device to explore options for luring and killing outdoor-biting malaria vectors: a report on design and field evaluation of the Mosquito Landing Box. Parasit Vectors.

